# Perspectives of Productive Aging: How Occupations Affect Health and Well-Being of Older Adults in Retirement

**DOI:** 10.1093/geroni/igaa057.1365

**Published:** 2020-12-16

**Authors:** Marilyn Cole, Karen C Macdonald

**Affiliations:** 1 Quinnipiac University, Stratford, Connecticut, United States; 2 Sacred Heart University, Bridgeport, Connecticut, United States

## Abstract

A predicted surge in the aging population presents a major challenge for public health in the USA. As occupational therapy researchers, we wondered how continued engagement in productive occupations affected the health and well-being of older Americans. Today’s retirees already understand the basics of successful aging, such as maintaining physical and mental fitness, and continuing an active lifestyle. Productive aging represents the next step: choosing roles and occupations that keep them engaged with others and their communities. As suggested by Clark (Jackson, Carlson, et al., 2012), occupations have the potential to provide a protective barrier to declining health, thereby lowering health care costs for the older population. Our Productive Aging qualitative studies (1 & 2) explore the lived experiences of life transitions, challenges, and adaptive skills implemented by participants (ages 65-80) in their own productive occupations. The findings of this follow-up study confirm and enhance themes of our first study (Cole & Macdonald, 2015): 1) prominence of the self-manager role, 2) intentionally maintaining social connections, and 3) engaging in self-fulfilling activities. Additional findings reflect current technology effects and elaborate how participants have met health challenges by adapting activities and environments (self-management), and using social resources (connections) to help them remain engaged in the occupations that give their lives meaning. Cole, M., & Macdonald, K. (2015). Productive aging: An occupational perspective. Thorofare, NJ: Slack, Inc. Clark, F., Jackson, Carlson, et al. (2012). Effectiveness of Lifestyle Intervention in promoting well-being of independently living older adults. Journal of Epidemiology and Community health.

